# Forecasting second-hand house prices in China using the GA-PSO-BP neural network model

**DOI:** 10.1371/journal.pone.0322821

**Published:** 2025-05-07

**Authors:** Jining Wang, Huabin Ji, Lei Wang

**Affiliations:** School of Economics and Management, Nanjing Tech University, Nanjing, China; University 20 Aout 1955 skikda, Algeria, ALGERIA

## Abstract

While the traditional genetic algorithms are capable of forecasting house prices, they often suffer from premature convergence, which adversely affects the reliability of the forecasts. To address this issue, the research employs a genetic-particle swarm optimization (GA-PSO) algorithm and develops a GA-PSO-BP neural network model through the integration of the BP neural network. Building upon this foundation, the study considers several pivotal factors affecting housing prices and employs a dataset comprising 1,824 transactions of second-hand homes from 2023 to 2024, gathered from Lianjia.com, to forecast housing prices in China. This work shows that the GA-PSO-BP neural network model demonstrates exceptional forecasting performance when dealing with complex and high-dimensional data, significantly minimizing forecasting errors. The test set achieved an RMSE of 0.786 and a MAPE of 8.9%. Its effectiveness in forecasting prices of second-hand houses notably surpasses that of a BP neural network model optimized by a single algorithm. This research provides more accurate forecasts of second-hand house prices in rapidly growing urban areas such as Guangzhou, thus providing essential insights for investors contemplating real estate investment.

## Section 1: Introduction

Real estate, a distinct commodity characterized by its long service life and robust durability, remains highly valued by consumers [1,2]. The report of the 20th Party Congress emphasized the general policy of “housing for living, not speculation”, with the goal of maintaining long-term stability and encouraging the healthy development of the market. Since 2023, several new policies have been introduced, from the central to local levels, to stabilize the economy and stimulate housing consumption, providing stronger policy support for real estate sector. Concurrently, with the advancement of urbanization, the tightening of land supply has led to escalating housing prices. In this context, the significance of second-hand homes in the real estate market has surged due to a notable increase in transaction volume [3]. Given that fluctuations in second-hand house prices can have direct impacts on individuals, households, financial markets, and urban development plans [4,5], the accurate forecasting of these prices has emerged as a critical focus of interest for both academic researchers and real estate professionals [6,7].

When forecasting the prices of second-hand houses, identifying the influencing factors amidst complex fluctuations is crucial for precise forecasting. On the macro level, economic indicators such as per capita savings, GDP per capita, and inflation rates significantly impact housing price variations. Additionally, human capital also exerts a positive influence on housing prices [8–11]. On the micro level, existing research indicates that the intrinsic characteristics of a house affect its price. For instance, studies have shown that the interaction between floor level and view positively influences housing prices [12]; smaller houses possess greater potential for appreciation and hence impact prices [13]; sunlight affects real estate pricing [14]; and the condition of a house can influence the likelihood of mortgage approval by lending institutions, thereby affecting prices [15]. Some scholars have considered the geographical space and location of a house, focusing on the spatial and distance factors influencing housing prices, though less attention has been given to the properties’ own characteristics [16]. With the evolution of behavioral economics, some researchers have begun to explore the impact of market sentiments and investor preferences on housing prices. On one hand, studies have found a positive correlation between housing sentiment and property prices [17]; on the other, speculative behaviors by buyers from outside the region also impact housing prices [18]. This article starts from the perspective of the house’s own characteristics, selecting factors such as type, size, orientation, renovation status, floor level, and price as the initial research variables.

After identifying the necessary factors for forecasting, finding an appropriate method for forecasting the prices of second-hand houses becomes particularly important. Recently, there has been a growing scholarly interest in the forecasting of second-hand house prices. Historically, autoregressive models were predominantly employed to estimate these prices [19]. With the development of technology, methods such as time series forecasting and machine learning have gradually become mainstream methods for forecasting second-hand house prices [20,21]. For example, Zhou et al. [22] analyzed second-hand house prices in Taipei City, utilizing a hybrid PSO-Bagging-ANN model that combines the Particle Swarm Optimization (PSO) algorithm with Artificial Neural Network (ANN). The results showed that this model surpasses the performance of support vector machines, classification and regression trees, and linear regression models in accuracy. Fang [23] used auction data from a provincial court system from 2018 to 2021, applying a combined genetic algorithm (GA) enhanced multilayer feed-forward neural network model for house price forecasting. The experiment showed that this model had a slightly superior forecasting impact compared to multiple linear regression and extreme learning machines, aligning more closely with real-world scenarios. Sun et al. [24] used data from Chongqing Municipality from 2000 to 2020 to boost the BP neural network model by combining GA and PSO algorithms, thus establishing a hybrid genetic particle swarm BP neural network model in order to forecast the value of pre-owned homes. The forecasting error of this model was approximately 0.5%, demonstrating its efficacy. However, the combined use of GA and PSO algorithms still encounters challenges related to local optima. Moreover, existing studies primarily focus on model performance and algorithm combinations without systematically summarizing the research gaps in this field. To address this limitation, we have provided a research gap analysis table in the S1 Table, which outlines the key findings of relevant literature and explicitly identifies the gaps our study aims to fill.

This study draws on existing research and combines the Genetic Algorithm (GA), known for its fast convergence rate and high precision, with the Particle Swarm Optimization (PSO), renowned for its effective localized search capabilities. First, the process begins with the encoding of individuals, followed by the application of genetic algorithm’s selection, crossover, and mutation operations to search for optimal candidates. These candidates are then incorporated into the PSO, with the refined individuals cyclically reintegrated into the GA. This iterative process enhances the BP neural network, resulting in the development of the GA-PSO-BP model, which forecasts second-hand house prices. This paper introduces the innovative GA-PSO-BP model, which integrates Genetic Algorithm (GA) and Particle Swarm Optimization (PSO) with a Backpropagation (BP) neural network, effectively mitigating the typical local optima issue in combined algorithms and displaying robust forecasting accuracy with complex, high-dimensional datasets.

The key innovations of this study are as follows: (1) To the best of our knowledge, this study is the first to apply the GA-PSO-BP model to the forecasting of second-hand house prices. While individual machine learning techniques such as BP, GA, PSO have been used separately for real estate price forecasting, their combined potential remains underexplored. Our research successfully integrates them into a hybrid framework for second-hand house prices forecasting, demonstrating its superiority over conventional approaches. The proposed model effectively addresses the local optima problem inherent in single-algorithm optimization methods, thereby significantly enhancing forecasting accuracy and robustness. (2) Another key innovation of this study is the meticulous data preprocessing and regularization techniques applied before model training. Unlike conventional studies that often rely on raw or minimally processed data, we implemented feature selection, normalization, and categorical encoding strategies to improve model performance. Feature selection was conducted using a combination of Recursive Feature Elimination (RFE) and Lasso regression, ensuring that only the most relevant variables were retained, thereby reducing dimensionality and enhancing interpretability. Additionally, normalization techniques were applied to mitigate the influence of magnitude discrepancies between variables, ensuring a more stable and efficient learning process. These preprocessing steps contribute to the novelty of our approach by optimizing data quality, which in turn improves forecasting accuracy. (3) This study goes beyond merely demonstrating the feasibility of the GA-PSO-BP model; it conducts comprehensive experimental evaluations to analyze the contributions of each algorithmic component. Through rigorous comparative experiments, we systematically assess the impact of each component on forecasting accuracy. Our findings provide detailed insights into how the hybrid GA-PSO-BP model outperforms traditional networks in terms of convergence speed, forecasting error reduction, and generalization ability. Moreover, we offer a transparent and reproducible methodology that can serve as a reference for future research. The in-depth analysis of individual algorithmic contributions is a unique aspect of this work, filling a gap in existing literature where such granular insights are often overlooked.

The structure of the paper is organized as follows: Section 2 displays the GA-PSO algorithm process. Section 3 discusses the processing of second-hand house price data and the construction of GA-PSO-BP neural network model. Section 4 verifies the effectiveness of the model through feasibility and quantitative analysis and examines the forecasting results. The results of this study are reviewed in Section 5.

## Section 2: GA-PSO-BP neural network modeling

### BP neural network

A feed-forward neural network with input, hidden, and output layers, the BP neural network integrates forward propagation with feedback mechanisms. The training process involves bidirectional propagation of feature parameter data. During forward propagation, the model generates outputs based on inputs, while during backpropagation, it contrasts the actual value with the forecasted ones. The network calculates loss and back-propagates it to update weights and biases, thus optimizing the parameters to enhance model performance. Due to its superior nonlinear processing capabilities, the BP neural network is commonly utilized in fields such as regression and forecasting [25,26]. Its efficacy in forecasting second-hand house prices is notably evident in two aspects: First, its potent nonlinear processing capability allows it to manage the various influencing factors dynamically, acknowledging their distinct impacts and the complex interrelations among them. This results in a nonlinear relationship between the price and each influencing factor, where the network’s adaptive and self-learning capabilities facilitate a more precise and realistic analysis. Second, its batch processing capability significantly streamlines the handling of large-scale forecasts, markedly reducing the need for human and material resources and improving forecasting efficiency.

**Fig 1:**
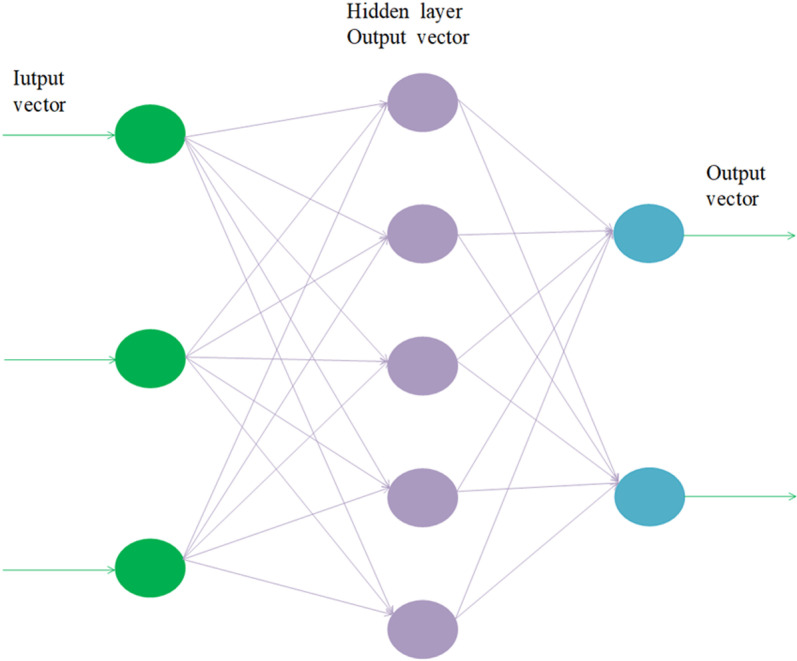
Neural network structure diagram.

Fig 1 shows the architecture of a BP neural network, in which three variables are represented by three input neurons, five by five hidden neurons, and two by two output neurons. The neural network architecture used in this paper is a single-output model with 30 hidden neurons and nine input neurons.

### Genetic particle swarm algorithm

#### Particle swarm algorithm (PSO).

PSO, a bio-inspired optimization algorithm, simulates the foraging process of birds. It is highly valued for its rapid convergence, minimal parameter requirements, and robust local search capabilities, making it widely applied across various domains such as feature selection and neural network training [27,28]. The algorithm initiates by randomly setting particle positions within the defined boundaries. With each iteration, the position and velocity of every swarm particle are renewed. The velocity and position updating formulas of PSO are as follows:


$$v_{i}^{t+1}=\left\{ \begin{array}{c} fix\left[v_{i}^{t}+c_{1}\cdot r_{1}\left(p_{z}^{t}-x_{i}^{t}\right)+c_{2}\cdot r2\left(p_{ig}^{t}-x_{i}^{t}\right)\right]\\ -v_{i}^{t}+2v_{i}^{t}\cdot rand \end{array}\right.\;\begin{array}{c} v_{i}^{t}\in \left[-v,v\right]\\ other \end{array}$$ (1)

$$x_{i}^{t+1}=\left\{ \begin{array}{c} fix\left[x_{i}^{t}+0.6v_{i}^{t+1}\right]\\ fix\left[3\cdot rand\right] \end{array}\right.\;\begin{array}{c} x_{i}^{t+1}\in \left[0,3\right]\\ other \end{array}$$ (2)

#### Genetic algorithm (GA).

Based on principles of genetics and natural selection, the Genetic Algorithm (GA) is an intelligent optimization algorithm noted for its superior global optimization capabilities and robustness in tackling complex optimization problems. Widely utilized in engineering optimizations, GA begins by initializing a population within defined constraints, where each individual is encoded as a fixed-length string using binary or real-number coding [29–31]. The process evaluates individual fitness, ranks them accordingly, and uses selection, crossover, and mutation operations to simulate the biological evolution process, producing a new generation of individuals. Through continuous evolution, the group reaches the best possible outcome, with the most fit individual being the final output. In contrast to the particle swarm algorithm, GA involves more complex coding, slower search speed, and higher computational complexity.

#### GA-PSO algorithm.

PSO algorithm is renowned for its proficient local search capability and low computational complexity. Conversely, the GA is recognized for its robust global search prowess. In recent years, the integration of GA and PSO has culminated in the development of the GA-PSO algorithm, which amalgamates the strengths of both to offer rapid convergence rates, high convergence accuracy, the ability to surmount local optima, and enhanced global search capability [32,33]. The GA-PSO algorithm enhances a synergistic interaction between the two algorithms, improving the search process and circumventing stagnation at local optima. In the GA-PSO algorithm, individual solutions are encoded followed by the application of genetic algorithm’s selection, crossover, and mutation processes to identify optimal solutions. These optimal individuals are then passed to the particle swarm algorithm, and the resulting individuals are fed back into the genetic algorithm, iterating this process. Lastly, the network architecture is updated with the optimized thresholds and weights.

This paper utilizes the GA-PSO algorithm to augment the performance of the BP neural network. The methodology entails: (1) Importing and preprocessing the training data, normalizing it to a specified range, and segmenting into a training and a test set. (2) Randomly initialize the parameters to construct the BP neural network architecture. (3) Utilizing the GA-PSO algorithm to encode the network parameters and initialize particle dynamics. The algorithm optimizes during the iteration process based on the set number of iteration rounds. The algorithm recalculates fitness metrics post-update of particle positions and velocities if the site ration cap is reached. During iterations, genetic operations such as selection, crossover, and mutation are executed as required, and the individual best positions of the particles are continually adjusted to align with the globally optimal configurations based on fitness evaluations. Upon reaching the iteration threshold, the optimal network parameters are derived. Subsequent training phases involve the backpropagation algorithm to update its parameters. After the set number of training rounds is completed, the entire process is finished. The integration of GA and PSO algorithms significantly enhances the initial parameter settings in the BP neural network, thereby boosting the model’s forecasting accuracy and overall applicability.

## Section 3: Forecasting analysis based on the GA-PSO-BP neural network model

### Data preprocessing and feature selection

The dataset used in this study consists of 1,824 transaction records of second-hand houses in the Baiyun District of Guangzhou City, spanning from 2023 to 2024, sourced from Lianjia.com. Table 1 illustrates a subset of this dataset. The large magnitude discrepancies within the dataset can adversely affect modeling results. Therefore, before constructing the model, the original dataset is normalized to eliminate the impact of magnitude differences. This paper applies normalization so that the data values fall within the range of (-1, 1).

**Table 1:** Some data examples of the data set.

Serial number	Housing type	Story	Area (square meters)	Orientation	Renovation	Housing prices (ten thousand yuan)
1	2 bedrooms, 1 living room, 1 kitchen, 1 bathroom	High floor	62.52	South	Hardcover	145
2	3 bedrooms, 2 living rooms, 1 kitchen, 1 bathroom	Medium floor	91.26	North and south	Hardcover	425
3	5 bedrooms, 2 living rooms, 1 kitchen, 2 bathrooms	Medium floor	262.37	South	Hardcover	2006.9
4	3 bedrooms, 2 living rooms, 1 kitchen, 1 bathroom	Low floor	114.15	North-northeast	Simple installation	599.8

The dataset presented in Table 1 includes a range of variables including housing type, area, orientation, renovation, story, and housing prices. The excessive inclusion of attributes pertaining to second-hand houses can interfere with forecasting accuracy. To address this, feature selection is employed to isolate the attributes that substantially influence the model’s results, while discarding those that are irrelevant or redundant, thereby facilitating dimensionality reduction, enhancing model performance, reducing computational time, and improving forecasting accuracy. In this paper, we employ recursive feature elimination from the wrapper method, alongside the Lasso regression model which incorporates a penalty term from the embedded method for feature selection. This method eliminates variables not jointly selected by both methods. Both experiments result in five critical factors: housing type, area, orientation, renovation, story, and housing prices, which are maintained as influencing factors in this study.

We executed a comprehensive series of statistical analyses to confirm the significance and pertinence of these attributes. To assess the relevance of the area variable, we calculated the Correlation Coefficient with the target outcome, which yielded a robust value of 0.87, indicative of a strong positive correlation. This finding underscores the pivotal role of area in influencing the outcome variable and substantiates its inclusion as a key attribute in our study. For the categorical variables, we applied Analysis of Variance (ANOVA), a statistical technique that assesses the mean differences across various groups to determine the presence of statistically significant disparities. In our case, ANOVA aids in determining the impact of different levels or categories of these variables on the target outcome. The results of the ANOVA tests substantiate these variables’ contributory significance to the research findings.

Table 2 reveals that all variables display relatively elevated *F*-statistics and minimal *p*-values. Specifically, a high *F*-statistic notably indicates that the means across different categories are substantially distinct from each other, while the minimal *p*-values substantiate that the differences are statistically significant. These findings suggest that the choice of housing type, orientation, renovation, and story are relevant attributes, given their significant impact on the dependent variable.

**Table 2:** Results of the ANOVA tests.

	Housing Type	Orientation	Renovation	Story
***F*-statistic**	2.75	4.12	2.96	3.98
***p*-values**	0.02	0.01	0.05	0.04

In summary, the robust correlation observed with the continuous variable (area) and the significant ANOVA results for the categorical variables substantiate the rationale for selecting these five attributes for our study. These attributes were chosen based on their significant associations with the target outcome, as indicated by both correlation and statistical significance analyses. After processing the gathered data, a total of 1,824 data samples were compiled. The data samples were subsequently randomized, with 70% allocated for training purposes and the remaining 30% designated for testing.

### GA-PSO-BP neural network model setup

#### Data output input settings and system parameter settings.

In the context of the GA-PSO-BP neural network model, this paper incorporates nine input nodes linked to five critical factors influencing house prices: house type, floor, area, house orientation, and decoration. It employs a single-output model with 30 hidden layers and one output layer to forecast second-hand house prices in Baiyun District, Guangzhou City, producing a solitary forecasted value. For the feature “housing type”, this paper introduces a nuanced approach by using four input values to capture subtle differences in property characteristics, thus reflecting their value; these values represent the number of rooms, kitchens, and bathrooms. For example, a “2 rooms, 1 hall, 1 kitchen, 1 bathroom” house would be encoded accordingly. For “area”, raw data are normalized within a [0,1] range to address scale discrepancies between different properties, ensuring that features contribute effectively without introducing scale bias during model training. For the feature “orientation”, since home buyers typically consider both “south” and “north” orientations, a binary indicator variable is employed to represent “south” and “north”, ensuring the encoding captures common buyer preferences. Consequently, if a single second-hand house may have both orientations, it is represented with two input values: [1, 1] for “south” and “north”, respectively, with each value taking 0 or 1 to denote absence or presence. The “renovation” feature is categorized into four levels — 0, 1, 2, and 3—corresponding to rough, simple, fine renovation, and others, respectively, to encapsulate the added value each level provides. The “story” feature is segmented into three categories—0, 1, or 2—to denote low, medium, or high relative height, thus enabling the model to account for the correlation between floor level and buyer preferences, as well as market valuations, where higher floors typically command a unique pricing approach. Finally, a 9-dimensional feature vector is obtained, combining all these features, with the house price as the target variable to be forecasted.

The specific operation is as follows: the first nine factors are used as input, and the final house price forecasting value is the output. The first 70% of the data is used as the training sample, resulting in an input vector represented as a 1276 × 9 matrix. During the training process, the GA-PSO algorithm optimizes the BP neural network’s performance by optimizing its weights and thresholds, thus improving the accuracy of the network model. The remaining 30% of the data is used as a validation sample to forecast house prices, from which the forecasted values are obtained. Simultaneously, BP, GA-BP, PSO-BP, and GA-PSO-BP models were used to forecast the values in the test set, and their forecasting results are comparatively evaluated. Subsequent analysis of the forecasting results focuses on discrepancies relative to actual figures. Table 3 illustrated the BP neural network’s parameters.

**Table 3:** Parameter settings of BP Neural Network.

Parameter name	Numerical value
Number of neurons in the input layer	9
Output layer neuron	1
Learning rate	0.01
Maximum number of training sessions	60,000
Target error setting	0.00001
Initial weights and thresholds	[-3,3]

Our neural network architecture in the research is structured into three layers: input, hidden, and output. The total quantity of neurons is set to nine in the input layer, 30 in the hidden layer, and one in the output layer. The learning rate is set to 0.01, with a maximum of 200 training epochs permitted. Due to the moderate size of the dataset, batch training is not applied. Before optimizing the particle swarm using a genetic algorithm, the initial weights and thresholds are predefined within the range of [-1,1].

#### Selection of the number of BP hidden layers and the number of nodes.

**Fig 2:**
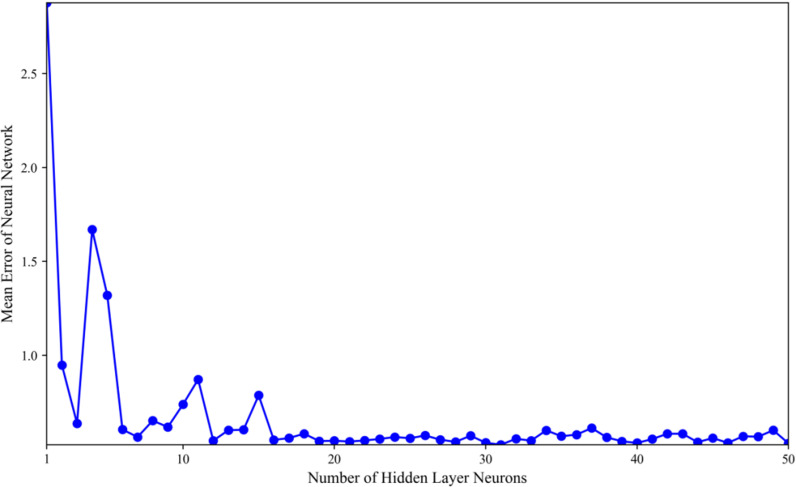
Neural network accuracy with different numbers of hidden layer neurons.

The predetermined configuration of input and output layers in the model contrasts with the optimal selection of hidden layer neurons in the BP neural network, which serves as a crucial parameter and optimization target for subsequent applications of BP, PSO, and GA-PSO in model optimization. A series of experiments were conducted to achieve an ideal balance between model complexity and training stability. By systematically varying the number of neurons in the hidden layer across a spectrum from smaller to larger configurations, the model underwent training through forward propagation and backpropagation algorithms, and the training data were utilized to fit the model. The model’s fitting performance is objectively assessed using a loss function, defined as the mean square error between the forecasted and actual house prices, where a smaller loss function value indicates higher accuracy and reduced forecasting error. In Fig 2 we can see the outcomes of the experiments.

Fig 2 presents that the number of hidden layer neurons significantly affects model performance. A configuration of 30 neurons in the hidden layer of the BP neural network achieves an optimal balance between model complexity and training stability. A low neuron count hampers the model’s learning capacity, inhibiting its ability to capture complex data features and, consequently, diminishing the accuracy of its forecasts. Conversely, increasing the neuron count enhances the BP neural network’s fitting capabilities, reducing model error incrementally. However, surpassing a certain neuron threshold introduces instability and potential training challenges, as excessive neurons may lead to overcomplex models that overfit noise in the training data, diminishing generalizability to new datasets.

Therefore, configuring the hidden layer with 30 neurons optimizes the BP neural network’s fitting performance while minimizing overfitting risks, ensuring stable convergence to a low error level.

#### GA-PSO-BP neural network modeling process.

**Fig 3:**
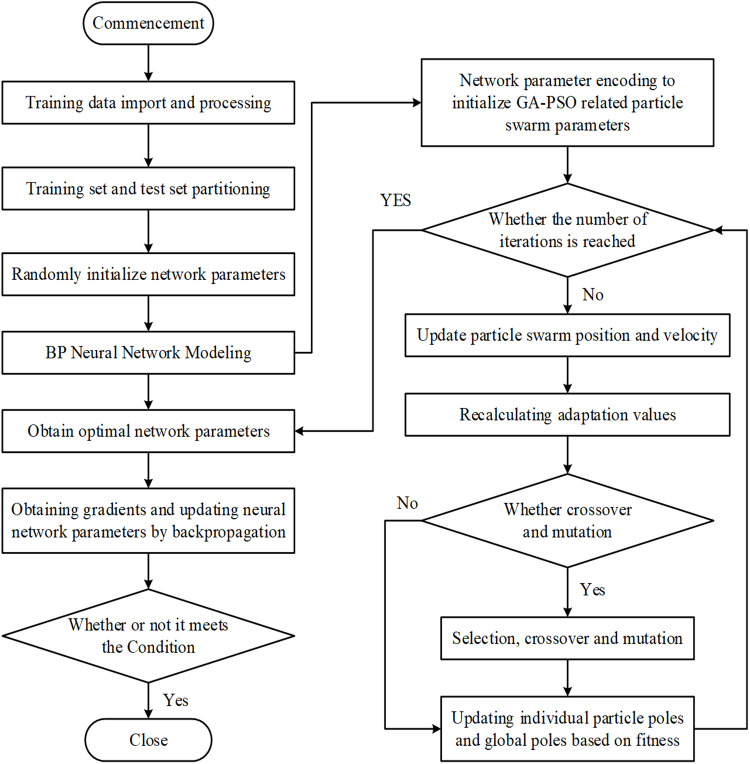
Flowchart of GA-PSO-BP neural network model.

The GA-PSO-BP neural network model’s flowchart, as presented in Fig 3, includes the following steps in detail:

Step 1: Code for each individual and initialization of the population. Each person was encoded using a real number, and the GA-PSO-BP neural network was used to forecast home prices using MATLAB software. Each encoding encompasses the complete set of BP neural network weights and thresholds, encoded with a length of S:


S=n×m+m×l+m+l (3)

This model considers the quantity of neurons in the input layer (*n*), the hidden layer’s number (*m*), and the output layer (*l*). We start with a population of 50 and set its size to initialize. Table 4 displays the definitions of the variables used in the GA-PSO-BP neural network model.

**Table 4:** Main variables of GA-PSO-BP neural network model and their meanings.

Notation	Hidden meaning	Notation	Hidden meaning
N	Number of particles	Pm	Probability of mutation
wmax&wmin	Maximum and minimum weights	c1,c2	Indicates acceleration constant (learning rate)
t	Current number of iterations	T	Maximum number of iterations
$x_{i}^{t}$	The position of particle i at the *t*-th iteration	$v_{i}^{t}$	Velocity of particle i at *t*-th iteration
$P_{g}$	The optimal fitness value for particle i	$P_{z}$	Optimal fitness value for the whole population
Pc	Crossover probability	$y_{i}$	Neural Network Forecasted Values

Step 2: Construct the fitness function. The fitness function, which is computed by determining the root mean square error (RMSE) for all training set samples, measures the overall forecasting error of the model. This function measures the deviation between forecasted and actual values, where a lower fitness value signifies superior forecasting accuracy of the model and reduced error.


$$error=\frac{1}{N_{t_{real}}}\sum \sqrt{{\left(t_{pred}-t_{real}\right)}^{2}}\frac{-b\pm \sqrt{b^{2}-4ac}}{2a}$$ (4)

Where $t_{pred}$ is the forecasted output of the neural network on the training data and $t_{real}$ is the actual training target data from the training set, where $N_{t_{real}}$ is the number of training set samples.

Step 3: Initialize the parameters of the particle swarm algorithm by setting the acceleration coefficients c1_and_ c2, defining the maximum and minimum weights wmax&wmin and specifying the initial velocity $v_{i}^{0}$.

Step 4: Based on the current number of iterations t, determine whether the maximum number of iterations T has been reached. If not, continue iteration.

Step 5: Update the position $x_{i}^{t}$ and velocity $v_{i}^{t}$ of each particle according to the current iteration, and recalculate the fitness based on the forecasted $y_{i}$ and actual values from the BP neural network.

Step 6: Selection, crossover and mutation operations are performed based on the crossover probability Pc and mutation probability Pm_, thereby updating the particle values as per the generated_ crossover and mutation operations.

Step 7: Compute the fitness value for each individual particle, identify the particle with the optimal fitness value $P_{g}$_,_ and the overall population optimal fitness value for the population $P_{z}$ through the mechanism of particle swarm algorithm.

Step 8: Continue repeating Steps 4–7 until the maximum iteration count is achieved. The final neural network parameters are obtained by retraining the BP neural network with the optimized weight thresholds.

In practice, we set the Genetic Algorithm (GA) population at 50, evolving over 100 generations. A mutation probability of 1% is established to preserve genetic diversity and prevent premature convergence. For the Particle Swarm Optimization (PSO) algorithm, a swarm of 50 particles iterates 100 times with an inertia weight of 0.8 to balance exploration and exploitation. The influence of the particles’ personal and global best positions is moderated by coefficients, each set at 0.5. The GA-PSO method integrates these parameters, merging GA’s global search capabilities with PSO’s efficient local optimization, ensuring a robust search process and avoiding local optima. Preliminary experiments validated the chosen parameters, consistently yielding high-quality solutions across multiple trials.

## Section 4: Analysis of experimental results

### Fitness curves for each optimization algorithm

**Fig 4:**
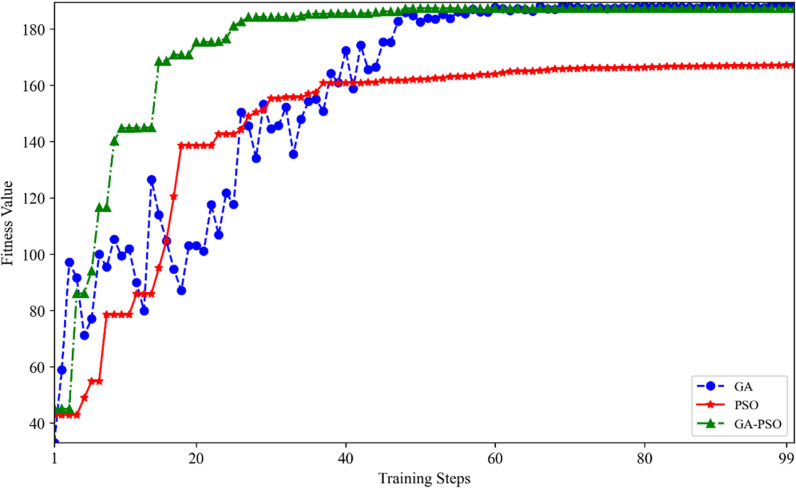
Iterative error profile.

To analyze the performance of the three algorithms GA, PSO, and GA-PSO, all employing identical initialization parameters and the highest possible number of cycles, the parameters of the BP neural network optimized by each algorithm are input into the backpropagation training. The fitness value for the comparison experiment corresponds to the inverse of the trained loss function value. The graph showing the algorithm’s adaptation with the number of iterations is presented in Fig 4.

As seen in Fig 4, the fitness values of all three algorithms increase with the number of iterations. GA and GA-PSO reach similar fitness values by the 45th iteration, which are higher than the fitness value of PSO at the 100th iteration. The convergence accuracies of GA and GA-PSO over PSO are notable. This superiority is attributed to the mechanics of PSO, where the adjustment of particle velocity and position is confined to the particle’s individual and neighboring experiences, potentially limiting the exploratory capability in expansive, high-dimensional complex problems. As a result, PSO is prone to entrapment in local optima, which hampers its ability to discover globally optimal solution, thereby yielding lower fitness values.

From Fig 4, it is also clear that PSO, GA, and GA-PSO reach their maximum fitness values at the 35th, 50th, and 20th iterations, respectively. Furthermore, PSO demonstrates a faster convergence speed compared to GA, while GA-PSO and GA exhibit the slowest convergence. This observation can be explained by GA’s comprehensive search capabilities through selection, crossover, and mutation, which, while beneficial for discovering superior solutions, tend to slow the convergence process. As previously mentioned, faster convergence can carry the possibility of too quickly settling into a local maximum.

Therefore, GA-PSO combines the benefits of both PSO and GA. It inherits the fast convergence characteristics of the PSO algorithm while increasing search diversity through GA’s selection and mutation mechanisms. This synergy enables the algorithm to swiftly identify optimal solutions while effectively circumventing local optima entrapments. GA-PSO can achieve a higher fitness value, similar to that of GA, within a shorter number of iterations, demonstrating superior performance in forecasting second-hand house prices. Forecasting such prices is inherently complex due to the multitude of influencing factors—such as location, size, amenities, and economic conditions—which are characterized by non-linear interdependencies. Traditional Backpro-pagation (BP) neural networks may struggle to capture these complexities, often resulting in slow convergence and susceptibility to suboptimal solutions. The GA-PSO hybrid method is particularly well-suited for this challenge as it optimizes weight initialization and finely tunes network parameters to enhance the BP model, thereby improving forecasting accuracy. Our study demonstrates that the GA-PSO-BP model strikes an optimal balance between computational efficiency and forecasting accuracy, making it highly effective in the dynamically evolving real estate market. A BP neural network optimization utilizing GA-PSO shows significant improvement in forecasting accuracy.

### Feasibility analysis of GA-PSO-BP neural network model

**Fig 5:**
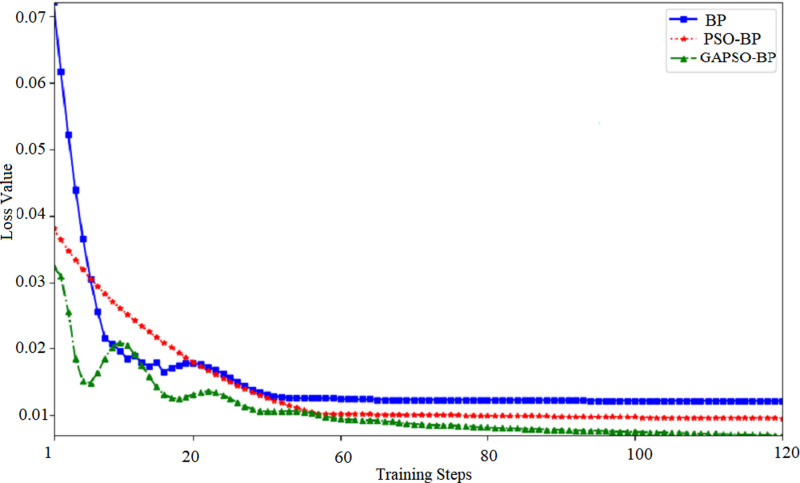
BP neural network training curve.

To verify the effectiveness of the GA-PSO algorithm in forecasting second-hand house prices and to establish its superiority over existing methods, this study conducts a thorough analysis of the performance of BP neural networks initialized by various methods throughout their training process. Given that both GA and GA-PSO achieve similar performance levels after sufficient iterations, this experiment primarily compares three BP neural networks with different initialization methods: random parameter initialization, initialization after PSO optimization, and initialization after GA-PSO optimization. Fig 5 displays the outcomes.

Fig 5 indicates that all three algorithms demonstrate a decrease in forecasting error as training progresses, but their performance differs significantly. The BP neural network with random parameter initialization exhibits a substantial forecasting error at the start of training. This initial high error can be attributed to the inadequacy of random parameters to effectively capture data patterns early in the training, thereby necessitating a prolonged period for parameter adjustment and error reduction. However, due to the BP neural network’s limited ability to search the solution space, even after extensive training, the final convergence error remains significant, making it difficult for random initialization to provide an effective initial parameter set.

Fig 5 illustrates that the BP neural network, when initialized with PSO-optimized initial parameters, exhibits a notably lower forecasting error compared with the network with random initialization at the start of training. The PSO algorithm capitalizes on the collective search experience of particles to find a more rational set of initial parameters, positioning the network nearer to the optimal global solution at the start of training. As part of the training, the PSO-optimized BP neural network converges rapidly, achieving a markedly lower final forecasting error than the network with random initialization. However, one of the main drawbacks of PSO is that its search process tends to settle at local optima, which can preclude the achievement of the global optimum. This issue is addressed by the GA-PSO algorithm. The BP neural network optimized by the GA-PSO algorithm performs similarly to the PSO-optimized network in the early stages of training, with lower forecasting error. The GA-PSO algorithm can efficiently optimize the initial parameters and continues to improve the model in the later stages of training, avoiding being trapped in local optima. The GA-PSO optimized BP neural network achieves a forecasting error of approximately 0.007, significantly outperforming the other two initialization methods. This underscores the GA-PSO’s efficacy in boosting the performance of the BP neural network’s performance in forecasting second-hand house prices.

### Quantitative analysis of the optimization algorithms

The performance of BP neural networks initialized with random parameters is juxtaposed against those initialized using three different optimization algorithms across training and test sets. This comparative analysis serves to delineate the strengths and weaknesses of each method. The numerical comparisons are shown in Table 5, which can be found here.

**Table 5:** Comparison of numerical results for each optimization algorithm.

Arithmetic	Root mean square error of the training set	Root mean square error of the test set	Mean absolute percentage error of the training set (%)	Mean absolute percentage error of the test set (%)	Number of iteration rounds when the algorithm converges
_**BP**_	1.211 (±0.16)	1.576 (±0.18)	15.3 (±2.19)	17.5 (±2.25)	–
**GA-BP**	0.712 (±0.10)	0.802 (±0.13)	9.8 (±1.61)	10.3 (±1.76)	50
**PSO-BP**	0.956 (±0.11)	1.021 (±0.30)	11.3 (±2.04)	12.7 (±1.93)	30
**GA-PSO-BP**	0.699 (±0.09)	0.786 (±0.11)	7.8 (±1.24)	8.9 (±1.02)	30

As Table 5 provided, the root mean square error (RMSE) of the BP neural network is 1.211 on the training set and 1.576 on the test set, with mean absolute percentage errors (MAPE) of 15.3% and 17.5%, respectively. Without optimization, the network struggles to learn and forecast complex data patterns, resulting in elevated error values. The GA-optimized BP neural network shows significant performance improvement, with RMSE reduced to 0.712 on the training set and 0.802 on the test set, and MAPEs decreased to 9.8% and 10.3%, respectively. Additionally, the GA-BP algorithm converged after 50 iterations. By emulating natural selection and genetic variation, GA effectively optimized the BP neural network’s initial parameters, enhancing data adaptation and markedly decreasing training and testing errors. However, a notable drawback of GA-BP is its slow convergence speed, requiring 50 iterations to reach a stable convergence state.

The PSO-optimized BP neural network has a root mean square error (RMSE) of 0.956 on the training set and 1.021 on the test set, with MAPE values improved to 11.3% and 12.7%, respectively. Although the PSO algorithm reduces the error of the BP neural network to some extent, its performance is not as effective as GA-BP. Notably, the PSO-BP reaches its optimal solution more rapidly, necessitating only 30 iterations. However, the higher testing errors suggest potential overfitting by the PSO algorithm or its tendency to fall into local optima, thus inadequately reducing forecasting errors.

The GA-PSO-optimized BP neural network demonstrated superior performance across all metrics. Specifically, the RMSE of the GA-PSO-BP model was 0.699 on the training set and 0.786 on the test set, with the MAPE values at 7.8% for the training set and 22.7% for the test set, both figures surpassing those of other algorithms. The observed trends in MAPE were consistent with those in RMSE, indicating that the GA-PSO algorithm not only effectively optimized the initial parameters of the BP neural network but also maintained low error values during both training and testing, thus enhancing the model’s forecasting accuracy. Meanwhile, the convergence speed of GA-PSO-BP was comparable to that of PSO-BP, requiring only 30 iterations to reach convergence, underscoring its potential as an effective optimization tool for BP neural networks in the task of house price forecasting.

### Forecasting experimental results

**Fig 6:**
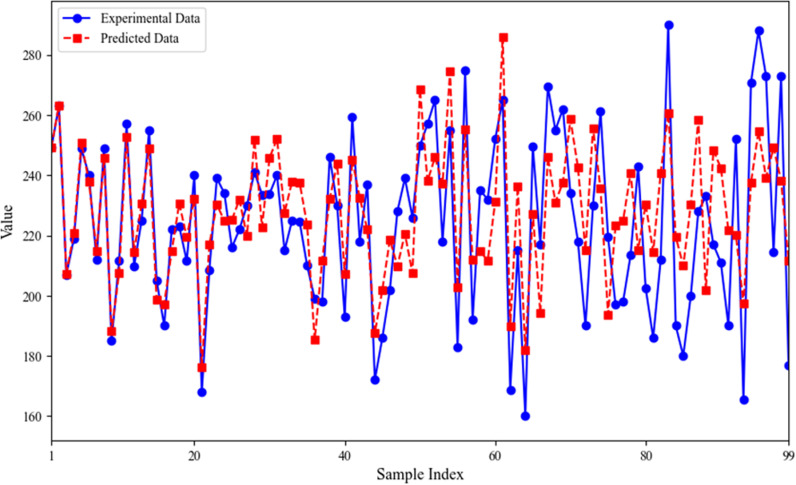
Scatter plot of forecasted experimental results.

To ensure the effectiveness of the suggested GA-PSO algorithm in the second-hand house price forecasting task, we first normalized the actual house price data of the samples, which served as the horizontal coordinate. Then, the BP neural network, maximized through the GA-PSO algorithm, was applied to guess these samples, with the forecasted values plotted as the vertical coordinate. The resulting curve is shown in Fig 6.

As seen from Fig 6, the correlation between the forecasted and actual values is strong, with several data points overlapping, indicating the absence of significant systematic bias in the model’s forecasting process. This suggests that the forecasted values are neither consistently overestimated nor underestimated, and the uniform distribution highlights the GA-PSO algorithm’s enhancement of the BP neural network’s forecasting performance, ensuring high accuracy and stability under varying data conditions. However, significant differences between the forecasted and actual values are observed at some data points, likely influenced by the impact of policy factors during specific periods, especially the large fluctuations in the real estate market between 2023 and 2024.

## Section 5: Conclusion

This study addressed the limitations of using a single algorithm for optimizing BP neural network forecasting of second-hand housing prices. In this paper, we first optimized the BP neural network with the help of the GA-PSO algorithm and then constructed the GA-PSO-BP neural network model. We then assessed the model’s utility in forecasting second-hand house prices through feasibility analysis and quantitative analysis. Finally, with the help of the second-hand house price data collected by Lianjia.com in the Baiyun District of Guangzhou, house price forecasts in China were generated by the model. Here are the key takeaways from the article:

(1) We applied the GA-PSO-BP model to the forecasting of second-hand house prices for the first time, successfully integrating BP, GA, and PSO into a hybrid framework. Through quantitative and feasibility analyses, this study demonstrated that the proposed model outperformed traditional methods.
(2) The Guangzhou second-hand housing data underwent preprocessing, including feature selection with RFE and Lasso regression to reduce dimensionality and normalization to ensure stability and efficiency. These steps enhanced data quality, improving market dynamics capture and forecasting accuracy. After rigorous training and simulation testing and validation, the model was able to more accurately capture the actual dynamics of the market.
(3) This study conducted a detailed experimental evaluation of the impact of each algorithmic component on forecasting accuracy. The results demonstrated that the integration of the GA-PSO algorithm with the BP neural network model enhanced forecasting accuracy and significantly reduced forecasting errors. Furthermore, this study provided a transparent and reproducible methodology, offering a valuable reference for future research exploring hybrid machine learning frameworks in real estate price forecasting.

These findings highlight the practical value of the GA-PSO-BP model, particularly in dynamic real estate markets. However, its performance may vary due to regional disparities in economic development levels, geographic characteristics, and infrastructure. In northern cities, factors such as heating facilities significantly impact housing prices, while in coastal cities, proximity to waterfront areas and climate resilience play a more dominant role. Similarly, in rapidly urbanizing second-tier cities, infrastructure expansion and migration trends exert greater influence on real estate market dynamics. To enhance the model’s cross-regional adaptability, regional characteristics can be integrated into the feature selection and training processes to address these regional disparities. In future studies, transfer learning techniques could be employed to fine-tune the model for specific urban contexts, ensuring optimal predictive performance even in markets with diverse economic and geographic features.

During the analysis process, it was found that sudden real estate policies could lead to extreme fluctuations in housing price trends, thereby increasing the difficulty of predicting second-hand housing prices. Although the model in this study focuses on housing-specific features, its high-precision predictive capability can still indirectly support policy formulation. By comparing changes in model prediction residuals before and after policy implementation, the disturbance intensity of policy shocks on market equilibrium prices can be quantified. Building on this, by further introducing market sentiment analysis—analyzing the emotional transmission effects following policy announcements from a behavioral finance perspective and incorporating sentiment variables as dynamic weights into the model—decision-makers can identify sensitive regulatory levers and develop differentiated intervention strategies without directly introducing policy variables. Future research will continue to optimize the model’s adaptability through such hybrid frameworks to balance prediction stability and policy relevance.

## Supporting information

S1 Table Research gap analysis table. (DOCX)

S1 File Striking image. (TIF)

## References

[1] Van Leuven AJ. Leveraging main street as a real estate amenity: downtown revitalization and residential property values. J Plan Educ Res. 2024;44(3):1488–502. doi: 10.1177/0739456X221082500

[2] Triveni G Danish F Efficient population mean estimation via stratified sampling with dual auxiliary information: a real estate perspective. Alex Eng J. 2024;104:680–7. doi: 10.1016/j.aej.2024.08.013

[3] Ren J, Gao X. Grid density algorithm-based second-hand housing transaction activity and spatio-temporal characterization: the case of Shenyang City, China. IJGI. 2024;13(8):286. doi: 10.3390/ijgi13080286

[4] Yin Z, Sun R, Bi Y. Spatial-temporal change trend analysis of second-hand house price in Hefei based on spatial network. Comput Intell Neurosci. 2022;2022:6848038. doi: 10.1155/2022/6848038 35655496 PMC9152383

[5] Pan Z, Liu Y, Wang H, Liu Y. How do house prices affect subjective wellbeing in urban China? Mediating effects of subjective socioeconomic status and household consumption. J Hous and the Built Environ. 2023;38(4):2559–80. doi: 10.1007/s10901-023-10053-x

[6] Li Y, Branco P, Zhang H. Imbalanced multimodal attention-based system for multiclass house price prediction. Mathematics. 2022;11(1):113. doi: 10.3390/math11010113

[7] Ji X, Ji X-F, Wei H, Chen Y, Xue W. BP network model based on SCLBOA for house price forecasting. Comput Intell Neurosci. 2022;2022:8148586. doi: 10.1155/2022/8148586 36275964 PMC9584679

[8] Duan J, Tian G, Yang L, Zhou T. Addressing the macroeconomic and hedonic determinants of housing prices in Beijing Metropolitan Area, China. Habitat Int. 2021;113:102374. doi: 10.1016/j.habitatint.2021.102374

[9] Liu M, Ma Q-P. Determinants of house prices in China: a panel-corrected regression approach. Ann Reg Sci. 2021;67(1):47–72. doi: 10.1007/s00168-020-01040-z

[10] Büchler SC, Niu D, Thompson AK, Zheng S. The impact of human capital and housing supply on urban growth. Urban Stud. 2023;61(2):214–30. doi: 10.1177/00420980231182074

[11] Xiao Y, Hui ECM, Wen H. Effects of floor level and landscape proximity on housing price: a hedonic analysis in Hangzhou, China. Habitat Int. 2019;87:11–26. doi: 10.1016/j.habitatint.2019.03.008

[12] Kang Y, Zhang F, Peng W, Gao S, Rao J, Duarte F, et al. Understanding house price appreciation using multi-source big geo-data and machine learning. Land Use Policy. 2021;111:104919. doi: 10.1016/j.landusepol.2020.104919

[13] Song Y, Ma X. Exploration of intelligent housing price forecasting based on the anchoring effect. Neural Comput Appl. 2023;36(5):2201–14. doi: 10.1007/s00521-023-08823-3

[14] Loro S, Lo Verso VRM, Fregonara E, Barreca A. Influence of daylight on real estate housing prices. A multiple regression model application in Turin. J Build Eng. 2024;96:110413. doi: 10.1016/j.jobe.2024.110413

[15] Millar MI, White RM. Do residential property assessed clean energy (PACE) financing programs affect local house price growth? J Environ Econ Manag. 2024;124:102936. doi: 10.1016/j.jeem.2024.102936

[16] Soltani A, Lee CL. The non-linear dynamics of South Australian regional housing markets: a machine learning approach. Appl Geogr. 2024;166:103248. doi: 10.1016/j.apgeog.2024.103248.

[17] Shen S, Zhao Y, Pang J. Local housing market sentiments and returns: evidence from China. J Real Estate Financ Econ (Dordr). 2022;68(3):1–35. doi: 10.1007/s11146-022-09933-w 38625289 PMC9716533

[18] Chinco A, Mayer C. Misinformed speculators and mispricing in the housing market. Rev Financ Stud. 2015;29(2):486–522. doi: 10.1093/rfs/hhv061

[19] Jin C, Lee G. Exploring spatiotemporal dynamics in a housing market using the spatial vector autoregressive Lasso: a case study of Seoul, Korea. Transactions in GIS. 2019;24(1):27–43. doi: 10.1111/tgis.12585

[20] Sanjar K, Bekhzod O, Kim J, Paul A, Kim J. Missing data imputation for geolocation-based price prediction using KNN–MCF method. IJGI. 2020;9(4):227. doi: 10.3390/ijgi9040227

[21] Zhan C, Liu Y, Wu Z, Zhao M, Chow TWS. A hybrid machine learning framework for forecasting house price. Exp Syst Appl. 2023;233:120981. doi: 10.1016/j.eswa.2023.120981

[22] Zhou W, Chen M, Yang Z, Song X. Real estate risk measurement and early warning based on PSO-SVM. Soc-Econ Plan Sci. 2021;77:101001. doi: 10.1016/j.seps.2020.101001

[23] Fang Y. Forecast of Foreclosure Property Market Trends during the Epidemic Based on GA-BP Neural Network. Sci Programming. 2022;2022:1–7. doi: 10.1155/2022/3220986

[24] Sun Z, Zhang J. Research on prediction of housing prices based on GA-PSO-BP neural network model: evidence from Chongqing, China. Int J Found Comput Sci. 2022;33(06n07):805–18. doi: 10.1142/s0129054122420163

[25] Wang Y, Liang S, John Xu Z-Q, Zhang T, Ji L. Artificial neural network aided unstable combustion state prediction and dominant chemical kinetic analysis. Chem Eng Sci. 2024;300:120567. doi: 10.1016/j.ces.2024.120567

[26] Zhang X, Zhang P, Yuan W, Hu S. Durability prediction of geopolymer mortar reinforced with nanoparticles and PVA fiber using particle swarm optimized BP neural network. Nanotechnology Reviews. 2024;13(1). doi: 10.1515/ntrev-2023-0214

[27] Wu W, Yao B, Huang J, Sun S, Zhang F, He Z, et al. Optimal temperature and humidity control for autonomous control system based on PSO‐BP neural networks. IET Control Theory & Appl. 2023;17(15):2097–109. doi: 10.1049/cth2.12467

[28] Hu D, Hu Y, Yi S, Liang X, Li Y, Yang X. Prediction method of surface settlement of rectangular pipe jacking tunnel based on improved PSO-BP neural network. Sci Rep. 2023;13(1):5512. doi: 10.1038/s41598-023-32189-0 37015985 PMC10073122

[29] Zhang C, Zhang M. Wavelet-based neural network with genetic algorithm optimization for generation prediction of PV plants. Energy Reports. 2022;8:10976–90. doi: 10.1016/j.egyr.2022.08.176

[30] Tian Z, Gan W, Zou X, Zhang Y, Gao W. Performance prediction of a cryogenic organic Rankine cycle based on back propagation neural network optimized by genetic algorithm. Energy. 2022;254:124027. doi: 10.1016/j.energy.2022.124027

[31] Lira JOB, Riella HG, Padoin N, Soares C. Computational fluid dynamics (CFD), artificial neural network (ANN) and genetic algorithm (GA) as a hybrid method for the analysis and optimization of micro-photocatalytic reactors: NOx abatement as a case study. Chem Eng J. 2022;431:133771. doi: 10.1016/j.cej.2021.133771

[32] Younespour M, Esmaelian M, Kianfar K. Optimizing the strategic and operational levels of demand-driven MRP using a hybrid GA-PSO algorithm. Comput Ind Eng. 2024;193:110306. doi: 10.1016/j.cie.2024.110306

[33] Zheng S, Pan Q, He D, Liu X. Reactor lightweight shielding optimization method based on parallel embedded genetic particle-swarm hybrid algorithm. Prog Nucl Energy. 2024;168:105040. doi: 10.1016/j.pnucene.2023.105040

