# Supplementary material for: Forecasting second-hand house prices in China using the GA-PSO-BP neural network model
Source: PLoS One. 2025 May 7;20(5):e0322821. doi: 10.1371/journal.pone.0322821 (PMC12057962; doi:10.1371/journal.pone.0322821)
Supplement: S1 Table — (DOCX) [file pone.0322821.s001.docx]

**Supporting information**

**S1 Table. Research gap analysis table.**

| **No.** | **Reference** | **Research Focus** | **Methodology** | **Key Findings** | **Limitations / Research Gap** | **How This Study Fills the Gap** |
| --- | --- | --- | --- | --- | --- | --- |
| 1 | Duan et al. [8] | Macroeconomic and hedonic influences on housing prices in Beijing | VAR Model and GWR Model | The VAR model elucidates the dynamic effects of macroeconomic indicators on housing prices, while the GWR model exposes the spatial disparities in the effects of hedonic properties. | This study does not account for the influence of housing characteristics on the prices of second-hand houses. | This research integrates factors related to housing characteristics into the prediction models for second-hand house prices, utilizing machine learning techniques for comprehensive analysis. |
| 2 | Xiao et al. [11] | Influence of floor level and proximity to landscapes on housing prices | Hedonic price model, regression analysis, and market segmentation | The correlation between floor level and housing prices exhibits a non-linear pattern, varying across multi-storey and high-rise buildings. | Most research presupposes a linear correlation between the floor level and housing prices. | It offers a nuanced examination of the non-linear effects of floor levels, accounts for variations across different types of buildings, and investigates the synergistic impacts of proximity to landscapes and floor levels on property values. |
| 3 | Kang et al. [12] | Prediction of house prices utilizing structural attributes and economic indicators | Data fusion framework using gradient boosting machine for feature extraction and geographically weighted regression | The proposed model demonstrates high predictive accuracy. Houses at lower price points and smaller sizes are more likely to appreciate in value. The inclusion of street view imagery and local amenities substantially influences appreciation rates. | Previous investigations have predominantly concentrated on the absolute prices of houses rather than on their appreciation rates. | The integration of diverse data sources, including street view imagery, property photographs, patterns of human mobility, and socioeconomic factors, enriches the analysis. |
| 4 | Song and Ma [13] | Integration of the anchoring effect with traditional determinants of housing prices | Hedonic Model 2SFCA Algorithm | Enhancing the model with a facility accessibility index augments the accuracy of price predictions. Integrating the anchoring effect within the price prediction framework elevates accuracy to 0.89. The ETR, RFR, and GBR models excel, achieving an accuracy rate of 0.9. | There is no consideration given to the enhancement of machine learning algorithms, which constrains the accuracy of predictions. | The GAPSO algorithm has been employed to refine the BP neural network, thereby enhancing the precision of second-hand housing price predictions in Guangzhou, with outcomes that more closely align with actual market conditions. |
| 5 | Loro et al. [14] | Examination of daylight's impact on real estate valuation in the Italian market | Simulation of 100 housing units assessing daylight metrics, followed by Hedonic Analysis and Multiple Regression Analysis | It has been established that Annual Sunlight Exposure (ASE) and Useful Daylight Illuminance (UDI) significantly impact housing prices, whereas Average Daylight Factor (ADF) and Spatial Daylight Autonomy (SDA) exhibit negligible influence. | The dataset used is relatively small (100 housing units), potentially limiting the generalizability of the results. | The study broadened the data set and thoroughly assessed how various housing characteristics influence price levels. |
| 6 | Millar and White [15] | Analysis of residential property assessed clean energy (PACE) financing programs and their effect on local housing price trends | Difference-in-differences regression analysis based on county-year data | PACE programs are associated with reduced home sales, decelerated price appreciation, and lower mortgage approval rates, impacting even non-PACE homes. | There is a lack of comprehensive analysis regarding how various types of renovations affect house prices. | Feature selection techniques were used to effectively encode housing renovation variables, improving the analysis of renovation impacts on property prices. |
| 7 | Soltani and Lee [16] | Application of advanced machine learning algorithms for regional housing price predictions in South Australia | Hedonic Modelling; Machine Learning; Multi-level Modelling | Machine learning models surpass traditional econometric approaches in capturing the complex non-linear dynamics prevalent in regional housing markets. | Consideration of factors related to housing renovations is absent. | Through ANOVA analysis, five principal factors that affect housing prices were identified, leading to a refined predictive model for property valuation. |
| 8 | Shen et al. [17] | Investigation into the effects of local housing sentiments on price dynamics in China | Partial Least Squares (PLS) | Local housing market sentiment in China is dynamically linked to fluctuations in housing prices. | This analysis does not account for sentiment-driven price dynamics within the Chinese housing market context. | The research delineates the influence of additional variables on property prices, identifying a research void that suggests a prospective avenue for future inquiries into how market sentiments interact with the dynamics of housing prices. |
| 9 | Chinco and Mayer [18] | Identification of general mispricing due to speculative or irrational behaviors in housing markets | Strategy Identification | The analysis primarily addresses general speculative behavior in the housing market without differentiating between types of buyers. | The influence of non-local buyers of second homes remains insufficiently explored. | This paper provides a comprehensive analysis of the second-hand housing market within the region, although it does not distinguish between the origins of the purchasers. |
| 10 | Zhou et al. [22] | Focus on real estate risk measurement and development of early warning systems | PSO-SVM | Evidence suggests that the PSO-SVM model offers superior performance in predicting real estate risk. | Although advanced machine learning techniques have proven effective in various real estate analyses, they have not been specifically utilized in second-hand housing price studies. | The application of a hybrid algorithm to the second-hand housing market analysis marks an innovative expansion of this methodological approach, offering deeper insights into the complexities of market dynamics in this sector. |
| 11 | Fang [23] | Predictive analysis of foreclosure market trends | GA-BP | The study emphasizes predictions of housing prices in foreclosure markets using foundational methods. | There is a restricted focus on sophisticated methods for price prediction in the foreclosure market. | This study employs a hybrid algorithm approach to real estate price prediction, effectively addressing the challenges of convergence seen in singular algorithm applications. |
| 12 | Sun and Zhang [24] | Strategies for predicting housing prices | GA-PSO-BP Neural Network | The GA-PSO-BP model, designed for predicting housing prices, has shown enhanced accuracy. | The study is limited by: 1. A narrow selection of variables, with inadequate attention to housing characteristics; 2. Basic optimization of algorithm combinations, lacking in-depth parameter adjustments; 3. Concentration on general housing price trends, without specificity for the rapidly evolving second-hand housing market. | This study refines GA-PSO-BP by integrating targeted feature engineering, optimizing parameters to improve convergence, and tailoring the model to second-hand housing markets in fast-growing cities like Guangzhou. |
| 13 | This study | Application of GA-PSO-BP neural network for predicting prices of second-hand houses | GA-PSO-BP Neural Network | This model effectively mitigates the local optimum issue in single-algorithm optimization by integrating BP, GA, and PSO into a hybrid framework. It considers the high-dimensional characteristics of the dataset and enhances predictive accuracy through rigorous data preprocessing, including feature selection with RFE and Lasso regression, as well as normalization for stability and efficiency. Through comprehensive experimental evaluation, this approach demonstrates superior performance in handling complex real estate data, significantly reducing prediction errors. As a result, it enables more precise forecasts of second-hand house prices in rapidly urbanizing cities such as Guangzhou, China, better capturing actual market dynamics. | The research examines the effects of intrinsic housing characteristics on second-hand house prices but overlooks the impacts of sudden policy shifts and other macroeconomic factors on overall housing price fluctuations. | Future research will integrate analysis of market sentiments, considering elements such as policy shifts and their impact on consumers, to further refine the accuracy of price predictions. |
