# Supplementary figures and images for: Forecasting second-hand house prices in China using the GA-PSO-BP neural network model
Source: PLoS One. 2025 May 7;20(5):e0322821. doi: 10.1371/journal.pone.0322821 (PMC12057962; doi:10.1371/journal.pone.0322821)

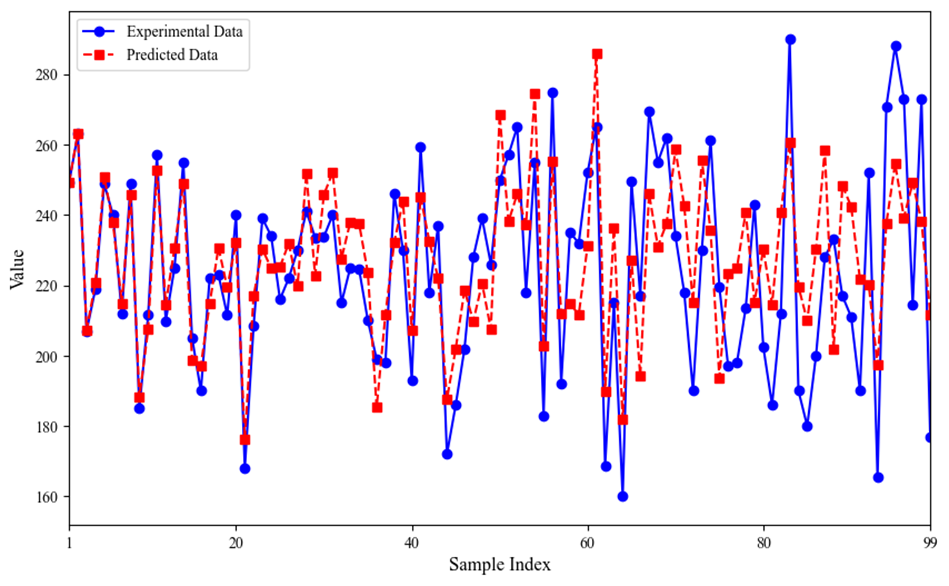

Supplement: S1 File — (TIF) [file pone.0322821.s002.tif]
